# University lecturers’ conceptions of online teaching in distance education courses in Vietnamese higher education

**DOI:** 10.1007/s10734-023-01058-0

**Published:** 2023-06-14

**Authors:** Uyen Nu Thuy Nguyen, David Kember

**Affiliations:** 1 Faculty of Education, College of Arts, Law and Education, University of Tasmania, Locked Bag 1340, Launceston, TAS 7250 Australia; 2 Launceston, Australia

**Keywords:** Conceptions of teaching, Online teaching, Distance education courses, University lecturers, Cultural influences

## Abstract

This study aimed to characterise academics’ conceptions of teaching in fully online undergraduate distance education courses with no on-campus component. The study aimed to fill a gap in the literature, as previous research had examined conceptions of teaching in face-to-face courses, with a few studies of blended teaching via the Internet in on-campus courses. Fourteen academics from five faculties in a Vietnamese regional university were interviewed, with the study taking place shortly after the outbreak of Covid-19. Grounded theory was used for data analysis. The results revealed four categories of conceptions of online teaching, namely online teaching (1) as transmitting structured knowledge and skills, (2) as guiding students to acquire knowledge and skills, (3) as facilitating students’ understanding via interaction and (4) as developing students’ understanding and capabilities. The four categories of conceptions were defined and distinguished by a set of six dimensions, which included e-Learning/LMS. The set of categories had some similarities to those found for face-to-face teaching, but also some distinctions which could be explained by the nature of online teaching and learning. The study, therefore, makes a major contribution by establishing a category scheme for conceptions of teaching in online distance education, with detailed characterisation of the four categories of conceptions. The descriptors of the conceptions showed cultural influences, particularly from a Confucian heritage, which is of significance as research into face-to-face conceptions had not found cultural variations.

## Introduction

Understanding teachers’ conceptions of teaching has been considered important for the enhancement of teaching quality as Kember (1997) and Postareff and Lindblom-Ylänne (2008) found that teachers’ conceptions strongly affected approaches to teaching in the face-to-face mode. Insights into teachers’ conceptions is valuable for explaining various behaviours, thus enabling a characterisation beyond these behaviours (Roberts, 2003), which may yield implications for measures to improve teaching quality. Similarly, Goodyear and Hativa (2002, p. 2) note, ‘knowing more about how teachers do and what they do is also a key to making educational research more relevant to their practice’.

There has been extensive research into teachers’ conceptions of face-to-face teaching. The outcomes of the body of research have been commonly presented as a set of conceptual categories of conceptions. The research topic reached a substantial body of results with sufficient levels of consistency for Kember (1997) to produce a review which synthesised a schematic representation of categories of conceptions of teaching. The representation featured two high-level orientations: teacher-centred/content-oriented and student-centred/learning-oriented. Each of the two orientations had two subsidiary conceptions. There was a fifth conception, labelled student-teacher interaction/apprenticeship, which was envisaged as intermediate between the two broad orientations. The over-arching features of the established categories of conceptions in these studies were found to be similar despite being labelled under different names.

The conceptions of face-to-face teaching were considered to be largely independent of context (Kember, 1997). The reviewed studies were conducted in a wide variety of Western contexts and Hong Kong. The current study adopted a grounded theory approach in order to examine whether there were contextual influences from either the Vietnamese cultural context or online distance education, which differs markedly from face-to-face on-campus teaching.

Other than the studies of face-to-face teaching, there have been studies which have examined teachers’ conceptions of teaching in blended courses or of their use of learning management systems (LMS). There has been a degree of divergence over the sets of categories which have been found in the studies that have taken place, limiting the value of any review aimed at developing an integrated synthesis of conceptions of teaching. These few studies revealed a range of conceptions that teachers held towards the use of websites or LMS in blended courses rather than the act of teaching online per se (Ellis et al., 2006; Lameras et al., 2008, 2012; McConnell & Zhao, 2006; Roberts, 2003).

The necessity of examining teachers’ conceptions of online teaching has been stressed more than ever as online education has increasingly gained its popularity especially after the Covid-19 health crisis (Bridges et al., 2023). Conducting specific research into conceptions of online teaching can also be justified as, while there is likely to be some relationship to conceptions of face-to-face teaching, there are also likely to be significant differences. Conceptions are deeply seated beliefs formed through relevant experiences. There will be relationships to face-to-face conceptions, since all teachers will have extensive experience of classroom teaching, and most will have taught face-to-face. There will also, though, be myriad other influences on the conceptions of online teachers, such as the technology-mediated nature of online teaching; accepted online teaching practices in the teacher’s university; and the nature of the Internet (Ellis et al., 2006). The study reported in this article is novel as it focuses on examining teachers’ conceptions of teaching in fully online courses, which would enrich the literature by adding insights into a substantial yet under-researched area in higher education.

The gap in the literature addressed by this study is that of there being a dearth of research into conceptions of teaching online distance education. This form of education has no on-campus classes as instruction takes place, synchronously or asynchronously, through learning management systems (LMS) or through online communication platforms, such as a Zoom. It is noted that while the studies in face-to-face conceptions did not show contextual variations, there have not been enough studies to determine if this is the case for conceptions of online teaching and learning. There is, then, ample justification for a carefully conducted study of online teaching to extend the understanding of these conceptions. Insights into these conceptions would help to explain how teachers teach online, which would be beneficial for improving the quality of teaching in online courses.

The present work used a grounded theory approach (Corbin & Strauss, 2015) to analyse the conceptions of online teaching of 14 Vietnamese university lecturers. The lecturers taught using three forms of online teaching: teaching through video recording for a centre of online teaching; teleconference teaching to remote partner centres; and teaching during the Covid-19 era, in which the lecturers adapted their face-to-face teaching for students unable to attend classes by teaching through videoconferencing platforms. As the study employed a grounded theory approach, this paper is structured based on the way the study evolved. Particularly, the “Literature Review” section focuses on delineating key concepts and reviewing significant studies into conceptions in blended and online teaching, rather than providing a comprehensive review as there is an extensive literature in conceptions of face-to-face teaching in higher education. Owing to the non-linear characteristics of grounded theory research, detailed engagement with the prior theoretical concepts and frameworks in related areas (e.g., the teaching and learning in higher education) will be explored and analysed where relevant in the “Discussion” section (Dunne, 2011).

The scope of the study is to examine conceptions of teaching in online distance education, which is defined as education which takes place entirely off-campus via an LMS or an online communication platform. The interviewees for the study were lecturers at a Vietnamese distance teaching university. The aims of the study are to (1) determine categories of teachers’ conceptions of teaching in online distance education; (2) characterise the categories of conceptions by a set of relevant dimensions related to teaching, learning and online learning technology; (3) compare the discovered categories of conceptions to those described in the literature for face-to-face teaching and on-campus teaching through blended learning using the Internet; and (4) determine whether the categories of conceptions of online teaching show any contextual influences from either Vietnamese culture or the online teaching medium.

## Literature review

Online education is often regarded as a subset of distance education and provides more flexibility in time and place compared to on-campus education (Anderson, 2011; Andrews & Tynan, 2012). Several terms have been used interchangeably to refer to online education such as e-Learning, virtual learning, blended learning, distance learning, online learning and online courses (Andrews & Tynan, 2012; Singh & Thurman, 2019). For the purposes of this paper, online education/ learning is referred to as a type of teaching and learning that is delivered in an online environment through the use of the internet and involves a geographical separation between students and teachers and the utilisation of some forms of technology for interaction between learners and tutors/instructors and between learners and learners (Anderson, 2011; Rapanta et al., 2020; Singh & Thurman, 2019; Yang & Tsai, 2017). Teaching during the Covid-19 era via videoconferencing mode bears important characteristics of online teaching.

Conceptions of teaching are generally understood as teachers’ underlying beliefs about teaching (Kember, 1997; Postareff & Lindblom-Ylänne, 2008). The terms conceptions and beliefs are used interchangeably in this paper. The body of research into conceptions of face-to-face teaching at university shared significant commonalities in the nature of conceptions found. Despite being presented under varying labels, these conceptions were generally categorised into two broad teacher-centred and student-centred orientations as posited in the review of Kember (1997) (see, for example, Akerlind, 2003; Samuelowicz & Bain, 2001). Orientations are understood as a broader level of categorisation, which includes two or more conceptions (Kember, 1997). In other words, conceptions are regarded as subordinate subcategories of the main categories—the orientations. Teacher-centred orientations were focused on the teachers and their delivery of a body of knowledge, while student-centred orientations emphasised on the students and their learning.

There are, notwithstanding, several dissimilarities among the studies as to whether (i) the conceptions were fairly stable (Kember, 1997) or relational in nature (Prosser & Trigwell, 1999) and (ii) the categories of conceptions were ordered in a continuum with the conceptions independent from each other (Kember, 1997) or in a hierarchy with higher-level conceptions comprising the lower ones (Akerlind, 2003; Prosser & Trigwell, 1999). Kember (1997) presented the categories of conceptions as discrete entities, which are demarcated from each other by several dimensions. Dimensions specify and add characteristics of the categories (Corbin & Strauss, 2015). These dimensions additionally illuminate the relationship between elements that form the categories. In the studies of Akerlind (2003) and Prosser and Trigwell (1999), categories are demonstrated as subcategories of hierarchically superior categories. Another difference was related to the existence of a transitional conception ‘student-teacher interaction’ that linked the two orientations identified by Kember (1997); however, no evidence of such a conception was reported in the study of Samuelowicz and Bain (2001).

There has also been research into conceptions of teaching in on-campus blended courses using the Web. In line with the findings from research into face-to-face teaching, the two teacher-centred and student-centred orientations were consistently observed across the range of studies (Ellis et al., 2006; Lameras et al., 2008, 2012; McConnell & Zhao, 2006; Roberts, 2003). The new aspects that these studies added into the literature are the new categories of conceptions related to the use of e-Learning system, which ranged from delivery of learning content to sustaining communication and promoting students’ learning. One common point of these studies is that they scrutinized the conceptions of blended teaching at campus-based universities. Very few studies into conceptions of teaching can be found in off-campus or online distance education setting both before and after the happening of the Covid-19 pandemic. Prior to the outbreak of the Covid-19, Gonzalez (2009) conducted a phenomenographic study to examine lecturers’ conceptions of teaching in postgraduate online distance courses. Using in-depth interviews, the study aimed to determine whether the previously developed frameworks for conceptions of and approaches to teaching as set out by Roberts (2003) and Kember and associates (Kember, 1997; Kember & Kwan, 2000) were applicable in an online distance setting. The results showed that the conceptions of face-to-face teaching developed by Kember (1997) were well suited to online teaching but those proposed by Roberts (2003) for teaching using the Web did not work. Gonzalez then suggested a model of conceptions of online teaching, comprising ‘the web for individual access to learning materials and information and for individual assessment’, ‘the web for learning related communication (asynchronous and/or synchronous)’ and ‘the web as a medium for networked learning’. We understand these conceptions to be more oriented to the use of e-Learning websites in these distance courses rather than to online teaching per se. Furthermore, this study employed a small sample (seven lecturers), which was not typical for phenomenography approach, in which fifteen participants are considered the minimum for revealing the variation in the research phenomenon (Trigwell, 2000). It is pertinent to note that the few studies of blended web-based teaching on-campus took place some time ago, which is of significance because of rapid technological developments in online and blended learning.

After the happening of the Covid-19, we observe that many studies into online teaching focus on teaching practices or strategies (Bridges et al., 2023). It has been identified in face-to-face teaching that teachers’ conceptions need to be considered to improve the quality of teaching and learning outcomes as how one teach is formed by their conceptions of teaching (Kember & Kwan, 2000; Postareff & Lindblom-Ylänne, 2008; Roberts, 2003). It is, therefore, necessary to extend the investigation of teachers’ conceptions into online teaching given the increasing prevalence of online education worldwide and the potential implications of research findings for improving online teaching quality. To our knowledge, this study has been the pioneer in examining lecturers’ conceptions of online teaching up to date. It should be noted that the current study differs from the research by Gonzalez (2009) in two ways: (i) this study is exploratory rather than based on predetermined frameworks; (ii) the online teaching examined involves both asynchronous and synchronous forms. These differences enable the exploration of more varied conceptions, which may be useful for refining or adding further dimensions or categories into the established model of conceptions of online teaching.

## Method

### Research approach

There have been two widely used approaches in prior studies about conceptions of teaching, namely phenomenography (Ellis et al., 2006; Gonzalez, 2009; Lameras et al., 2008, 2012; Trigwell & Prosser, 1996) and grounded theory (Kember & Kwan, 2000; Lam & Kember, 2006; Samuelowicz & Bain, 2001), or a combination of both, i.e. phenomenographic stance for interviews and grounded theory for data analysis (McConnell & Zhao, 2006; Roberts, 2003). Kinnunen and Simon (2012) have identified several similarities of these two approaches. First, semi-structured interviews with purposive sampling are often used for data collection. Second, new themes and categories emerge from analysis process, rather than predicating on the existing theory. The analysis process is iterative in nature involving reading transcripts, coding themes or categories, refining these categories, and creating connections between these categories. The largest difference between the two methods lies in the research aims. Whereas phenomenographic research focuses on discovering variations in people’s conceptions or experiences, grounded theory emphasises the actions of people besides understanding the variations in their conceptions. It aims at formulating a theory or model that ‘has to show action and change, or the reasons for little or minimal change’ (Strauss & Corbin, 1990, p. 123).

Grounded theory was chosen for the study reported in this article. This study is a part of a wider project aimed to investigate teachers’ conceptions of online teaching, how they translated their conceptions into their teaching approaches and the alignment between their conceptions and teaching approaches. The utilisation of grounded theory would offer an in-depth understanding of not only teachers’ conceptions of how they should teach but also their actions/teaching in practice and pertinent factors influencing this process. The outcome is not purely limited to the categorisation of different teaching conceptions or approaches but to uncovering the meaning including the influence of contextual elements underlying these conceptions and approaches.

### Data collection

The research was conducted at a Vietnamese regional university. This university is well-known for its online distance courses and one of the pioneers in implementing blended and online teaching for full-time courses in Vietnam. In contrast to other universities which were originally set up for full-time teaching, this university has implemented online education for off-campus students at its center of online teaching since 2006. The university also switched to online teaching for on-campus students at its member universities in response to the Covid-19 pandemic in 2020.

In alignment with the tenets of grounded theory approach, the study used purposive sampling for the interview. Lecturers who started with face-to-face teaching for on-campus courses and subsequently moved to online teaching for the same courses for off-campus students were invited to participate. This could allow us to explore the transition these lecturers have made in their teaching conceptions when they shifted from face-to-face to online teaching. Another aim of purposive sampling was to include lecturers who were representative as far as possible in terms of rank, years of teaching, disciplinary background, with different levels of responsibility, and professional experience. The lecturers from the five faculties, namely Faculty of English, Faculty of Business Administration, Faculty of Law, Faculty of Economics and Faculty of Banking, were selected as the online distance programmes offered by the university belong to the disciplines under these faculties. The tentative number was 20 lecturers, which is similar to the scale of research conducted by previous researchers in area of teachers’ conceptions of teaching (Kember & Kwan, 2000; McConnell & Zhao, 2006; Roberts, 2003). However, the data became saturated after the interviews with 14 lecturers. These lecturers had a wide range of experience in teaching adults off-campus and adolescent full-time students in face-to-face mode, varying from 7 to 42 years of teaching.

The study employed semi-structured interviews with open-ended questions. The interviews were constructivist in orientation when both the interviewer and interviewees were together constructing data (Merriam, 2009). The interview was conducted individually, face-to-face, and the participants chose their preferred language of interview, which is either in English or Vietnamese to ensure that they could freely express themselves. Open-ended questions were developed from key concepts and themes from the literature and from the first author’s experience as a practitioner were used for the interview. Since teachers’ beliefs and values cannot be explicitly stated, much of the questioning was indirect. For instance, when asking about teachers’ conceptions of teaching, indirect questions such as ‘What are your aims in doing online teaching? How do you define your role in online teaching?’ were used. The interview guide was constructed in relevance to the questions underpinning this research and participants’ responses acted as a source for subsequent questions. The interviews, lasting from 20 to 110 min, were recorded and transcribed verbatim. All the transcripts were returned to the interviewees for verification.

### Data analysis

The analysis process adhered to the guidelines of grounded theory approach (Corbin & Strauss, 2015). The first author made notes, transcribed and did preliminary analysis after the first and second interviews. The analysis revealed that the online teaching at the university was conducted in both asynchronous form, video recording, and synchronous form, teleconference teaching for online distance courses and videoconferencing teaching for full-time students, during the Covid-19 era. The two authors then discussed modifying the interview questions to elicit more information on the online teaching at distance courses at the university. The interviewer also found out that the participants often compared their online teaching for distance courses with that for on-campus students and liked to discuss their online teaching during the Covid-19 era. At the time the data relevant to online distance courses at the center of online teaching became saturated, we decided to expand our examination into online teaching during the Covid-19 era though the research originally intended to merely focus on examining online distance courses. The discussion between the authors happened between the interview rounds to refocus the interview guide to emerging aspects.

After the data collection was completed, a list of initial codes or concepts was generated. These codes were later added, removed and refined through a process of reading the transcripts over several times, finding similarities and differences between the formulated concepts and the new data. This process helped to condense the large volume of data to nine provisional categories. These categories were refined through a process of comparing and contrasting and we finally agreed on four main categories of conceptions of online teaching. We did rounds of collective discussion to agree on the dimensions, which helped to delimit these categories. This process ceased when each of the categories was fully developed with sufficient dimensions to characterise these categories. The final stage involved establishing a schema which captured the essence of the examined phenomenon—conceptions of online teaching. The process of analysing data was iterative with constant and multiple comparisons and contrasts, which is a distinctive feature of grounded theory method (Corbin & Strauss, 2015). NVivo 21 was used to manage the data.

## Results

### Categorisation of conceptions of online teaching

**Fig. 1:**
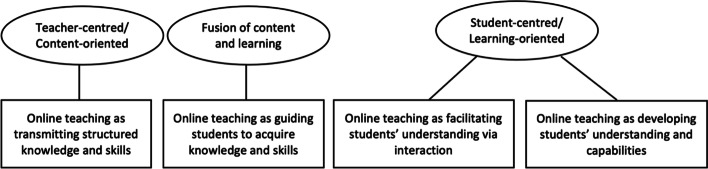
Categorisation model of conceptions of online teaching

The results revealed four categories of conceptions of online teaching as displayed in Fig. 1. These conceptions were grouped into three major orientations. The first orientation was teacher-centered, and emphasised delivering the body of knowledge and skills defined in the curriculum. The second orientation placed importance on teachers’ engagement with orienting students to the learning process. The third orientation was student-centered, which foregrounded the development of students’ understanding and capabilities and comprised two subordinate conceptions.

The four categories of conceptions of online teaching are distinguished by some dimensions revealing different relationships between the teacher and the student, the use of e-Learning system, the content and knowledge as shown in Table 1. Table 2 presents the exemplar quotations for these dimensions, which represent the totality of the concept. The sections that follow discuss each of these categories in turn.

**Table 1:** Dimensions used to delimit conceptions of online teaching

Dimension	Online teaching as transmitting structured knowledge and skills	Online teaching as guiding students to acquire knowledge and skills	Online teaching as facilitating students’ understanding via interaction	Online teaching as developing students’ understanding and capabilities
Teacher	Designer, presenter	Observer, tutor	Facilitator	Facilitator, promoter
Teaching	Transmit knowledge and skills	Guide students to sources of knowledge and skills	Interact to help students understand and learn	Help students to grow intellectually and personally
e-Learning/LMS	For storing studying resources	For self-study and individual assessment	For asynchronous communication and feedback provision	For stimulating thinking and learning application
Student	Passive recipients	Active recipients	Knowledge co-constructor	Active creator
Content	Defined by curriculum and organised by teachers	Defined by teachers	Constructed by students within teachers’ framework	Constructed by teachers and students
Knowledge	Possessed by teachers	Delimited by teachers	Constructed by students	Discovered by students

**Table 2:** Exemplar quotations for each of the categories

Categories	Representative quotations
Online teaching as transmitting structured knowledge and skills	I need to select the core content, systematise essential concepts of that chapter to help students achieve the learning outcomes of that course (BM3) Teachers need to master the expertise and knowledge of the subject they are teaching (BM1) You [teachers] need to have an attractive presentation style (ENG3) I mostly lecture in online teaching, so the quality of the lessons mainly depends on my careful preparation (BM3) This [LMS] is for students to get access to resources, like the syllabus, reading materials, some news for forums activities (ENG2)
Online teaching as guiding students to acquire knowledge and skills	Teachers mainly instruct and orient learners to seek for knowledge and information. Furthermore, teachers need to teach students research and self-study skills (LAW3). Teachers can, to some extent, have a certain influence on students’ learning so that they can go on the right track (LAW1) We have to be well-dressed and look so gay, fresh, and energetic. We should show them our passion, our energy at the very beginning of the lessons (ENG2) Lecturers have changed their teaching methods to engage and motivate students and also trigger their self-study abilities. Especially, learners should form their own opinions about any certain matter (BM1) Teachers can provide students with further resources to promote their self-study, self-practice … and design online learning activities in the e-Learning system (ENG1)
Online teaching as facilitating students’ understanding via interaction	Pedagogically, an effective lesson involves interactivity, engagement, and interest for learners … A teacher needs to know how to organise interactive activities instead of talking all the time … Learners need to have opportunities to share, discuss, solve problems, and learn from each other (BM2) Teachers and learners have equal role in the process of learning. We are both responsible for building the lessons. I do not advocate one-sided way of teaching in which teachers are the centre … I learn from my students in my teaching (LAW1). The students said that they had the feeling that they were having real interaction with their friends and teachers in real-time online classes … I think that is an important part of good online teaching. I mean the interaction between learners and learners, between learners and teachers (ENG4) It would be better if I could get involved in the interactive activities in the e-Learning website… I can create forum, chats and everybody can share their answers and I can give them feedback. In this way, teachers and students can have a closer relationship with each other (ENG3)
Online teaching as developing students’ understanding and capabilities	I want my learners to analyse, assess the situations and be creative with their knowledge. I also want my learners to apply the knowledge and skills acquired to their working lives, especially for adult learners … If they know to think critically and evaluate their work, they can be creative and work more effectively (BM2) Even though learners might not work in this field in the future, their knowledge about law will be useful in their lives. When legal matters arise in their daily lives, they will know how to solve them (LAW2) When you step into any school or university in Vietnam, you can see a big poster with the statement: “One has to learn manners before letters”. Manners refer to good morals and letters means expertise or talent. As Uncle Ho^a ^said, “a person with talent but without morals is useless. Otherwise, a person with good conduct but without talent cannot do anything”… The purpose of tertiary education is teaching not only expertise but also conduct or ethics of doing a profession. Learners need to be well-rounded citizens to work effectively upon their graduation (BM2)

^a^Also known as Ho Chi Minh. He was the first President of the Democratic Republic of Vietnam

#### Online teaching as transmitting structured knowledge and skills

Teachers who held this conception viewed online teaching as communicating the essential body of knowledge prescribed in the course objectives to students. Teachers selected the nuts and bolts of a subject, arranged them into coherent lessons and presented these lessons. They acted as a ‘sage on the stage’ and used their power to regulate students’ behaviour in online classes. Profound academic knowledge was regarded as an important attribute of a teacher. Another quality that was perceived important was teachers’ ability to present online effectively or their ‘stage performance’. Lecturing was the major teaching method. Students were regarded as playing the role of passive recipients. They were seen to be akin to sponges who absorbed the knowledge teachers presented to them either in online class or in the LMS without questioning the veracity of the knowledge or having opportunities to join in creating the online learning resources. The lecturers considered the LMS to be a repository of studying materials and resources and a medium for information delivery.

#### Online teaching as guiding students to acquire knowledge and skills

In this conception, the focus was gradually shifted from teachers to students. Online teaching was viewed as accompanying and orienting students to sources of knowledge and skills. The lecturers still played a significant role though they did not hold the absolute power as in the conception of knowledge transmission. Interestingly, it seemed that the definition of the right knowledge or effective studying method was largely decided by teachers. Providing support in academic and non-academic forms was regarded as one of the most important features of online teaching. The academic support related to teachers signifying the knowledge to be learnt and directing students to information sources. Non-academic support was connected to building students’ motivation. Teachers believed that they should display a good model in terms of attitudes since this would influence students’ interest and engagement in online learning. The lecturers acknowledged students’ role in constructing knowledge; however, there was little evidence in whether they took any further action regarding the knowledge that students created. Students were viewed as active recipients of knowledge, exercising their abilities and activeness to achieve the targeted outcomes. Their role was expanded compared with that in the knowledge transmission conception, yet within certain limits defined by the lecturers. The LMS was regarded as a place for self-study and individual assessment, besides storing information. They defined the areas of knowledge to be learnt and evaluated on the LMS.

#### Online teaching as facilitating students’ understanding via interaction

Online teaching was viewed as an interactive process between teachers and students and among students themselves and aimed at building students’ understanding. The lecturers emphasised the necessity of maintaining interaction in synchronous online classes for checking students’ understanding, creating opportunities for them to express opinions and providing feedback to learners. The lecturers viewed their roles as ‘guide on the side’ or being equal to students, appreciated students’ contribution and acted as a co-constructor of knowledge with students. Interaction was believed to be essential as it provided a feeling of teachers’ and learners’ presence in online environment, which increased understanding for students and motivated the teaching and learning process. The LMS was considered to be useful for asynchronous communication and providing feedback to students.

#### Online teaching as developing students’ understanding and capabilities

This conception advanced from ensuring and building students’ understanding to expanding and connecting their understanding to real-world tasks. Online teaching focused on enhancing students’ understanding at a more sophisticated level, involving thinking critically, evaluating situations, solving problems and expressing creativity. The second facet related to enhancing students’ ability to apply their knowledge and skills to solving meaningful tasks and gaining hands-on experience. The third facet envisaged the holistic development of students by teaching them good conduct and ethics, which served their professions and their lives. Interestingly, the role of e-Learning website was almost invisible in this conception. The data implied that the lecturers took it for granted that the role of technology was to stimulate thinking and learning application.

### Classification of lecturers to conceptions of online teaching

The respondents were found to hold multiple conceptions of online teaching and move back and forth in the continuum of conceptions. The predominant conception of online teaching, held by eleven lecturers, was in the orientation focusing on both content and learning, followed by the teacher-centred conception expressed by ten lecturers. The least prevalent category with five responses belonged to the student-centered orientation, which focused on the development of students’ understanding and capabilities. There was a significant divide between the group of lecturers under the conception category ‘online teaching as transmitting structured knowledge and skills’ and the group under the category ‘online teaching as developing students’ understanding and capabilities’. Those holding the former conception did not show any evidence of the latter and vice versa, except for one participant (BM2) who expressed views compatible with both conceptions.

## Discussion

The most significant outcome of this research is the categorisation of conceptions of online teaching, enabling a comprehensive understanding of teachers’ conceptions in various teaching environments and contexts. This study has been, up to the time of writing, one of the few empirical studies that established the categories of conceptions of teaching in fully online courses. The categories of conceptions of online teaching were viewed as an ordered set of qualitatively distinct conceptualisations and progressed in terms of sophistication. Each category was independent from the others and occupied a position within a continuum of conceptions. The quotations of the higher-level conceptions did not comprise components of the lower-level ones in this sample of lecturers. Table 3 summarises and shows the gradual development of conceptions of teaching across different environments including face-to-face, blended and fully online from the pre-existing studies to the current one.

**Table 3:** Conceptions of face-to-face teaching and conceptions of blended and online teaching

	Kember (1997)	Samuelowicz and Bain (2001)	Roberts (2003)	McConnell and Zhao (2006)	Ellis et al. (2006)	Lameras et al. (2008)	Gonzalez (2009)	Lameras et al. (2012)	The current study
Conceptions focused on teacher or content	Imparting information	Imparting information	Web for subject information	eLearning or network learning or resource-based learning as a medium for uploading packaged materials	Blended learning as replacing the responsibility of being a teacher	VLE as a means of providing access to, or clarification of, content.		VLE as a means of supporting information transfer and recall	
	Transmitting structured knowledge	Transmitting structured knowledge	Web for individual and independent self-paced learning and assessment		Blended learning as providing students with information		The web for individual access to learning materials and information, and for individual assessment		Online teaching as transmitting structured knowledge and skills
		Providing and facilitating understanding						VLE as a means of supporting application and clarification of concepts	
Transitional Conception	Student-teacher interaction/ apprenticeship		Web for group work analysis, decision and making dialogue						Online teaching as guiding students to acquire knowledge and skills
Conceptions focused on student or learning	Facilitating understanding	Helping students develop expertise	Web for real-time dialogue and learning		Blended learning as developing student understanding through aligning media to intended learning outcomes	VLE for assessment, feedback provision and interaction between teacher and student to enable students’ understanding of concepts	The web for learning related communication (asynchronous/syn chronous)	VLE as a means of supporting development and exchange of ideas, and resource exploration and sharing	Online teaching as facilitating students’ understanding via interaction
		Preventing misunderstandings							
	Conceptual change/ Intellectual development	Negotiating meaning	Web for asynchronous dialogue and reflective learning		Blended learning as helping students develop and apply new concepts	VLE for supporting personal meaning-making through social negotiation to enable developing of concepts	The web as a medium for networked learning	VLE as a means of supporting collaborative knowledge-creation and development of process awareness and skills	Online teaching as developing students’ understanding and capabilities
		Encouraging knowledge creation							

The over-arching features of the conceptions discovered in this study display a degree of relationship to those identified with respect to face-to-face teaching (see Akerlind, 2003; Kember, 1997; Samuelowicz & Bain, 2001) and the studies that examined conceptions of teaching in blended teaching (e.g. Ellis et al., 2006; Gonzalez, 2009; Lameras et al., 2008; Roberts, 2003), which served to validate our findings. The respondents were found to hold multiple conceptions (cf. Kember, 1997; Norton et al., 2005) and sometimes shifted across these conceptions in response to external requirements. The findings reflect the relational nature of the conceptions of online teaching, which supports the results of Prosser and Trigwell (1999). The lecturers with student-centred belief at times need to rely on teacher-centred pedagogies to build blocks of fundamental knowledge for students before engaging them into higher-level studying activities. The lecturers’ conceptions can also be influenced by external constraints such as the assessment requirements of accredited courses at the university. Unavoidably, they need to teach to the test to some extent, prompting them to switch to teacher-centred conceptions. One more point of note is that the divide between the group of lecturers under two online teaching orientations illuminates the existence of a boundary between two extremes of teaching conceptions, which echoes the findings from a study of Prosser et al. (1994, p. 228), showing ‘two strongly contrasting subsets’ of conceptions in face-to-face teaching: one with heavy focus on the teaching activity and other with students as the focus.

On the other hand, the study discovered several distinctive aspects in the conceptions of online teaching. First, the study found no evidence of the conception ‘imparting information’ as identified in the former studies. The participants repeatedly emphasised the significance of organising and systematising essential knowledge of the course and arranging them in a logical manner instead of merely disseminating a large amount of information to learners. A possible explanation is that fully online distance courses need to be properly structured in teaching activities and learning content in the LMS, which could help to offset the absence of direct face-to-face interaction. The prior planning and preparation of the online teaching materials give teachers the opportunity to carefully plan instructional material to cater for self-study. We suspect that the conception of ‘imparting information’ might not exist in the fully online teaching mode. To date, there have been very few studies investigating teachers’ conceptions of online teaching; hence, further empirical research needs to be conducted to corroborate this finding.

Second, a transitionary category has been established under the orientation of ‘a fusion of content and learning’, which was not found in the previous studies that examined conceptions of teaching in blended and online teaching except for the study of Roberts (2003). This ‘in-between’ bridging conception was found to be dominant among the lecturers in this study’s settings. The finding is likely to portray a gradual shift in teachers’ conceptions from teacher-centered to student-centered orientation, which might occur as a consequence of the tension between contrasting educational beliefs that co-exist in Vietnam. On the one hand, deeply entrenched cultural values associated with Confucian philosophy which centers the teachers in the learning process still have a pronounced effect on the Vietnamese educational system (Nguyen, 2017; Pham, 2013). The majority of participants positioned themselves in an authoritative status and identified their roles as the transmitter of knowledge. This issue is further compounded by an over-emphasis on disciplinary theories and insufficient focus on practice components in the higher educational curriculum of Vietnam (Tran et al., 2014) and the assessment approach focusing on single-end product (Thanh, 2011). These situations inescapably put teachers in the position of knowledge transmitters and learners as passive recipients. On the other hand, the fact of increasingly globalised economies in the twenty-first century has prompted the Vietnamese educational system to transform its teaching and learning practices to keep up with the world’s changes. Educational reforms have been launched to adopt Western constructivist pedagogies, seeking a marked shift in roles of teachers and students and teaching and learning pedagogies.

Teachers need to be the ‘facilitator of learning, the nurturer of creative thinking’, and learners should be ‘active, practical, flexible and creative agents’ (Tran et al., 2014, p. 105). These policies are inherently at odds with the long-established belief upholding the central role of teachers. It appears that teachers are caught between these two opposing views and over-burdened with different responsibilities they are supposed to perform. They need to ensure that students can obtain the targeted knowledge and skills prescribed in the curriculum while at the same time adopt a student-centred role. The conception focusing on both content and learning could be the most appropriate way to resolve these contradictions. There is a degree of erosion in the authoritative position of teachers and knowledge towards facilitating students’ autonomy and learning. This finding corroborates the result of a former study into Vietnamese teachers’ views of adopting the Western constructivist approach (Nguyen & Hall, 2017), which stated that ‘the old images and attitudes persisted alongside a cautious interest in exploring the new ideas about teaching and new models of the teacher’ (p. 251).

The third difference is concerned with the position of e-Learning-related conception. In this study, the LMS was believed to be useful for ‘self-study and individual assessment’ in the intermediate conception of ‘online teaching as guiding students to acquire knowledge and skills’. In the previous studies, these e-Learning-related conceptions such as ‘web for individual and independent self-paced learning and assessment’ (Roberts, 2003) and ‘web for individual access to learning materials and information and for individual assessment’ (Gonzalez, 2009) were included in the subject/information focused orientation by Roberts and Gonzalez. We argue that the transitional orientation should be a more relevant and suitable position for this conception. While the lecturers construct the studying activities in the e-Learning system, students can use the website in their own time, study at their own pace and reflect on their progress via assessment activities.

Finally, several cultural and contextual facets of the conceptions of online teaching were discovered. This result is not in agreement with Kember’s (1997) review of conceptions of face-to-face teaching, which concluded that categories of conceptions were internationally applicable. The difference is likely to be due to the studies encompassed by the Kember’s review. The only Asian sample was from Hong Kong, where, at the time, universities had a strong international perspective. The lecturers in this study perceived the importance of exercising their power to monitor discipline and students’ behaviour in online classes. They reminded students to focus on the lessons when students showed signs of inattentiveness or engaged in off-task behaviours. This aspect was not seen in closely related studies from other contexts. The underpinning reason could be that the Vietnamese educational system is strongly imbued with Confucian values, which uphold the superior status of teachers (Pham, 2008). Confucius stated that teachers and students ought to behave in a manner that suits their roles and position, and students need to show deference and obedience to their teachers (Lee, 1996). In the Vietnamese context, students’ inattentiveness or off-task behaviours are normally interpreted as disrespectful to teachers. It is not uncommon for teachers to use their authority to deal with these situations. This deeply entrenched cultural belief is usually prevalent in traditional face-to-face lectures. It is interesting to notice that this perception is also transferred to the online teaching and learning environment.

Furthermore, the interviewees indicated that they should demonstrate a good model for students. This belief is also a continuation of Confucian standards that teachers should set good examples of, not only professional knowledge, but also impeccable morals (Pham, 2008). Another closely related concept in Vietnamese culture worth discussing is ‘face’ or the good image of a person. Maintaining face is particularly significant in the teaching profession since it is viewed as ‘the noblest career among noble careers’ in Vietnamese society. The factors contributing to one’s ‘face’ include internal moral qualities and external expressions such as dressing style or behaviors (Nguyen, 2015). This is clearly articulated by the academics when they said that they should display good attitude, show their passion or dress smartly in their online classes.

Another interesting finding is that the lecturers believed that online teaching should aim to enhance students’ well-rounded development in not only intellectuality but also behaviours and ethics. This conceptualisation is unique to the study’s context and can be viewed as a culturally rooted belief. A similar finding is recorded in a study into Vietnamese teachers’ conceptions of the purposes of higher education (Nguyen & Kevin, 2016). Teachers in this study believed that, together with academic competence, ‘morals’, which encompasses work ethics, personality and behaviours, are essential purposes of Vietnamese tertiary education. Graduates need to be taught how to perform in their work and to act with good manners such as behaving politely with adults and teachers and respecting others and behaving appropriately towards the community. On the whole, this conception is distinctive for the way it emphasises students’ holistic growth. Rich elements including knowledge and skills application and capabilities and personality development are all incorporated in the conception.

## Conclusion

The study is of research into conceptions of online teaching in the context of an online distance education course. This distinguishes the work from previous research into conceptions of teaching, which has almost all been of courses taught wholly on-campus, or predominantly on-campus with a degree of Internet component or blended learning. Its most significant finding is the characterisation of four categories of online teaching comprising: ‘online teaching as transmitting structured knowledge and skills’; ‘online teaching as guiding students to acquire knowledge and skills’; ‘online teaching as facilitating students’ understanding via interaction’; and ‘online teaching as developing students’ understanding and capabilities’. These categories are viewed as distinct conceptualisations and ordered within a developmental continuum. They are distinguished from each other by six dimensions, demonstrating the relationship between the teacher and student, the use of an e-Learning system, the content and knowledge.

The descriptors of the categories revealed influences from the teaching and learning environment of online distance education. There were also signs of cultural influences on the descriptors of the categories of conceptions, which is of interest as the research into conceptions of face-to-face teaching had not revealed contextual variations. The fact that this study was conducted in a country undergoing a transitioning process with competing existence from traditional Confucian values and new Western ideas might lead to the formation of a transitional conception and its predominance among the participants.

The research is intended as a precursor to further research into how conceptions of online teaching influence approaches to teaching. This future research should be of significance as conceptions of face-to-face teaching have been found to have a strong relationship to teaching approaches and this has had implications for teaching quality enhancement. It will be of interest to see whether there is also a relationship between conceptions and approaches when teaching is through the online medium.
